# The Effects of G-CSF on Proliferation of Mouse Myocardial Microvascular Endothelial Cells

**DOI:** 10.3390/ijms12021306

**Published:** 2011-02-22

**Authors:** Jiming Li, Yunzeng Zou, Junbo Ge, Daifu Zhang, Aili Guan, Jian Wu, Lei Li

**Affiliations:** 1 Department of Cardiology, Shanghai East Hospital, Tongji University School of Medicine, Shanghai, 150 Jimo Road, Shanghai 200120, China; E-Mails: lijmsh912@yahoo.com.cn (J.L.); dfzhangsh912@yahoo.com.cn (D.Z.); 2 Department of Cardiology, Zhongshan Hospital, Fudan University, Shanghai Institute of Cardiovascular Diseases, Shanghai 200032, China; E-Mails: alguansh912@yahoo.com.cn (A.G.); jwush912@yahoo.com.cn (J.W.); llish912@yahoo.com.cn (L.L.); 3 Institutes of Biomedical Scienses, Fudan University, Shanghai 200032, China

**Keywords:** myocardial microvascular endothelial cell, G-SCF, Hypoxia inducible factor 1, p53 mRNA, HIF-1 mRNA

## Abstract

This paper explores the effect of granulocyte colony-stimulating factor (G-CSF) on mouse myocardial microvascular endothelial cell (CMECs) proliferation. CMECs were harvested from C57/BL6 mice. CMECs were cultured in medium containing G-CSF (0 ng/mL, 20 ng/mL, 40 ng/mL, 60 ng/mL) for five days. Proliferative activity of CMECs was examined by CCK-8 method. Hypoxia inducible factor-1 (HIF-1) and p53 expression levels was determined from the mRNA obtained by reverse transcription polymerase chain reaction (RT-PCR). Results showed that the purity quotient of the CMECs, which were cultured by the method of modified myocardial tissue explant culture, was higher than 95%. Compared with control untreated cells, the proliferative activity of CMECs and the expression level of HIF-1 mRNA in these cells were enhanced by G-CSF treatment, whereas the expression level of p53 mRNA was markedly reduced. It may be concluded that G-CSF could promote the proliferative activity of CMECs, which might be mediated by upregulation of HIF-1 and downregulation of p53.

## 1. Introduction

Granulocyte colony-stimulating factor (G-CSF) is a hormone produced by the body that stimulates the bone marrow to produce more white blood cells. G-CSF is also produced as a drug. Studies have found that G-CSF injections reduce the severity and duration of neutropenia in patients with some types of cancer. G-CSF has also been applied to therapeutic angiogenesis induction in animal models [1–5] and humans [6–11]. The mechanisms for the angiogenic effects of G-CSF are partly attributable to increased numbers of myelomonocytic cells in the circulation and their release of VEGF, in addition to the mobilization of endothelial progenitor cells [12]. Cardiac angiogenesis is crucially involved in the adaptive mechanism of cardiac hypertrophy and p53 accumulation is essential for the transition from cardiac hypertrophy to heart failure. Pressure overload initially promoted vascular growth in the heart by hypoxia-inducible factor-1 (Hif-1)-dependent induction of angiogenic factors, and inhibition of angiogenesis prevented the development of cardiac hypertrophy and induced systolic dysfunction. Sustained pressure overload induced an accumulation of p53 that inhibited Hif-1 activity and thereby impaired cardiac angiogenesis and systolic function. These results indicate that the anti-angiogenic property of p53 may have a crucial function in the transition from cardiac hypertrophy to heart failure [13]. We suppose that G-CSF may stimulate myocardial microvascular endothelial cell proliferation through modulating HIF-1 and p53 expression.

In the present study, we cultured a mouse myocardium microvascular endothelial cell line (CMECs) and sought to examine the effect of G-CSF on CMEC proliferation. Then, we investigated the mechanism of G-CSF in promoting CMEC proliferation and angiogenesis through examining the effect of G-CSF on p53 and HIF-1 mRNA expression. Our study provides a new view and theoretical basis for clinical therapy of heart disease.

## 2. Materials and Methods

### 2.1. Materials

Culture medium, fetal calf serum (FCS), glutamine and antibiotics were from Gibco BRL (Paisley, UK); EC growth supplement, gelatin, trypsin, amphotericin B and hydrocortisone were from Sigma (Poole, UK); RNA-tocDNA Kit, Avidin Biotin Complex were purchased from Nanjing JianChen bioengineering institute. Dulbecco’s modified Eagle’s medium (DMEM) was purchased from Invitrogen, Carlsbad, CA, USA. RNAzol B was purchased from Biogenesis Ltd (Bournemouth, UK); Mouse murine leukemia virus reverse transcriptase was purchased from Gibco BRL (Gaithersburg, MD, USA). A Cell Counting Kit-8 was purchased from Dojindo Laboratories (Kumamoto, Japan).

### 2.2. Mice Myocardical Microvascular Endothelial Cell Cultures

Hearts were excised from 10–12-week-old mice (*n* = 44) under aseptic conditions. Cardiac ventricles were cleaned by sterilized Hank’s solution and then cut into pieces. A homogenate of ventricles was suspended and filtered by sequential 200 μm and 60 μm microfiltration. The filtered cells were cultured in culture flasks (75 cm^2^) at 37 °C in a humidified atmosphere with 5% CO_2_ in endothelial cell growth medium (ECGM), as supplied by the manufacturer, supplemented with 10 ng/mL human recombinant epidermal growth factor, 1 μg/mL hydrocortisone, 50 μg/mL gentamicin, 50 μg/mL amphotericin B, 12 μg bovine brain extract and 2% v/v fetal calf serum (FCS) for three days.

### 2.3. Proliferation Assay

The CMECs were plated in collagen-coated 24-well plates (10^4^ cells/well) and cultured in DMEM growth medium. Then, different concentrations of G-CSF (control, 20 ng/mL, 40 ng/mL, 60 ng/mL) were added to the cells and the cells were incubated. Cell viability and proliferation assays were performed by using the Cell Counting Kit-8 (CCK8; Dojindo, Kumamoto, Japan) or trypan blue staining, according to the manufacturer’s protocol. Briefly, after treatment, a 10 μL of CCK-8 solution was added to each well. After incubation at 37 °C for 2 h in a humidified CO_2_ incubator, absorbance at 450 nm was monitored with the SpectraMax Plus 384 microplate reader (Molecular Devices, Sunnyvale, CA, USA). It should be 1000–1500 cells/100 μL when culturing cells. After 5 days of incubation, it will be 2000–3000 cells/100 μL.

### 2.4. Reverse Transcription and Polymerase Chain Reaction (RT-PCR)

Total RNA samples were prepared from cultured CMECs (2–3 days) in exponential phase using the TRIzol reagent (Invitrogen), according to the manufacturer’s protocol. To eliminate genomic DNA, 10 μg of total RNA was treated with TurboDNase (Ambion) for 30 min at 37 °C. RNA integrity and quality were assessed by electrophoresis, and 1 μg of the DNase-treated RNA was then reverse transcribed into cDNA using a High Capacity RNA-tocDNA Kit (Applied Biosystems). The following gene-specific primers were used: GAPDH-F: 5′-GGAAAGCTGTGGCGTGATGG-3', GAPDH-R: 5′-GTAGGCCATGAGGTCCACCA-3′; p53-F: 5′-GCTGAGTATCTGGACGACA-3′, p53-R: 5′-CAGGCACAAACACGAACC-3′; HIF-1-F: 5′-GTGATGGACAGGGTATGG-3′, HIF-1-R: 5′-GGAGAAGAGCGGTTGTAT-3′. Each cycle consisted of denaturation (30 s at 94 °C), annealing (30 s at 60 °C), and elongation (30 s at 72 °C). PCR products were subjected to 2% agarose gel electrophoresis.

### 2.5. Statistics

All data are presented as mean ± standard error (SE). The standard unpaired Student’s t-test was used to analyze significant differences among the data.

## 3. Results

### 3.1. CMECs Morphology and Identification

**Figure 1.**
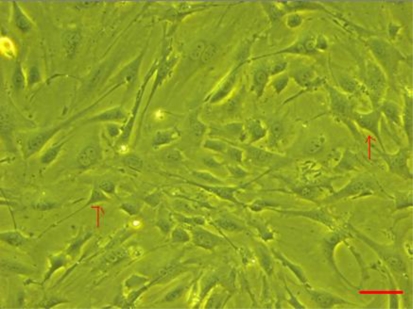
Myocardium microvascular endothelial cells (scale bar: 60 μm, 200×).

Endothelial cells were present around myocardium tissue after two days of incubation. These cells exhibited a megagon or spindle shape. On the second and third day, more and more endothelial cells were observed around the myocardium tissue mass. After removing the tissue mass, endothelial cells were cultured for another 2–3 days. These cells displayed a megagon or spindle shape or rather an elongated shape (Figure 1).

**Figure 2.**
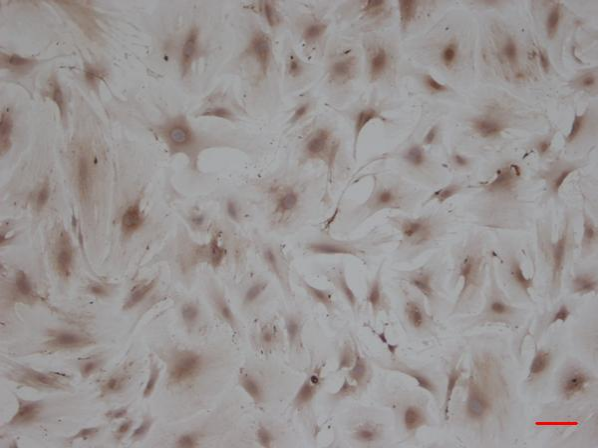
Immunocytochemistry Avidin Biotin Complex staining of myocardium microvascular endothelial cells (scale bar: 120 μm, 100×).

The endothelial cell phenotype was confirmed by acetylated LDL uptake, Factor VIII-Rag immunocytochemical staining. Results showed that the purity of CMECs was higher than 95% (Figure 2).

### 3.2. CMEC Proliferative Capacity

**Figure 3.**
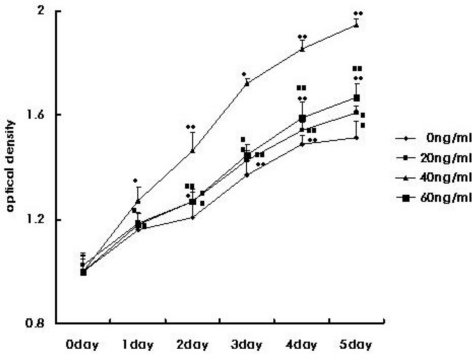
Myocardium microvascular endothelial cell proliferation. Data are mean ±SEM from ten independent experiments and each experiment was an average of triplicate measures. * *P* < 0.05, ** *P* < 0.01, compared with control (0 ng/mL G-CSF); ^##^ *P* < 0.01, compared with group (40 ng/mL G-CSF).

The proliferative capacity of CMECs in medium containing G-CSF was significantly (*P* < 0.05; *P* < 0.01) higher than in control medium. At 40 ng/mL G-CSF, the proliferative capacity was highest. Significant statistical difference (*P* < 0.05; *P* < 0.01) was observed between groups (Figure 3).

### 3.3. HIF-1 and p53 mRNA Expression

**Figure 4.**
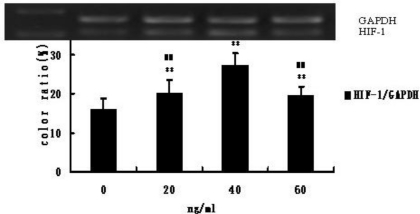
HIF-1 mRNA expression. ** *P* < 0.01, compared with control; ^##^ *P* < 0.01, compared with group (40 ng/mL). Top panel: RT-PCR analysis of mRNA expression of HIF-1 and GAPDH control; bottom panel: Quantification is the ratio of gray scale in band 1 (HIF-1) to gray scale in band 2 (GAPDH). Quantification for HIF-1.

Compared with control cells, the HIF-1 mRNA expression level of CMECs in medium containing G-CSF was markedly (*P* < 0.01) increased (Figure 4). The HIF-1 mRNA expression level was the highest at 40 ng/mL G-CSF, and was significantly (*P* < 0.01) higher than that in the other two treatment groups (20 ng/mL and 60 ng/mL) (Figure 4).

**Figure 5.**
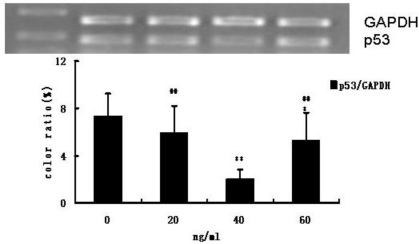
p53 mRNA expression. ** *P* < 0.01, compared with control; ^##^ *P* < 0.01, compared with group (40 ng/mL). Top panel: RT-PCR analysis of mRNA expression of p53 and GAPDH control; bottom panel: Quantification is the ratio of gray scale in band 1 (p53) to gray scale in band 2 (GAPDH). Quantification for p53.

Compared with the control cells, the p53 mRNA expression level of CMECs in medium containing G-CSF was markedly (*P* < 0.01) decreased (Figure 5). At 40 ng/mL G-CSF, p53 mRNA expression level was lowest. p53 expression level observed in the group with 40 ng/mL G-CSF treatment was significantly (*P* < 0.01) lower than that in other two groups (20 ng/mL and 60 ng/mL) (Figure 5).

## 4. Discussion

Previous findings have demonstrated the safety and feasibility of G-CSF treatment in patients suffering from late revascularized STEMI in a prospective, randomized, placebo-controlled analysis. G-CSF was not superior to placebo regarding improvement of global as well as regional myocardial function [14]. Another trial demonstrated significant increase of myocardial perfusion in the short-term. Several stem-cell populations, which are considered to improve myocardial regeneration or neovascularization, are significantly mobilized by G-CSF [15].

Angiogenesis, the formation of new capillaries from preexisting vessels, plays an essential role in development and pathological conditions such as wound healing, tumor growth and metastasis, and revascularization of the myocardium following myocardial infarction (MI) [16]. Formation of new blood vessels is critical for supplying the healing infarcted myocardium with oxygen and nutrients to sustain metabolism. Myocardial angiogenesis and improved myocardial function are keys to cure cardiac disease. Therefore, to obtain pure microvascular endothelial cells, it is important to establish a microvascular endothelial cell culture system. Isolation and culture of microvascular endothelial cells has been difficult due mainly to small initial yields and overgrowth of contaminating cells [17]. Therefore, in previous studies, endothelial cells from large vessels, e.g. human umbilical venous or aorta endothelial cells, were often used [18,19]. Because of obvious organ and tissue specificity, it is difficult to perform a more objective and accurate evaluation of pathological changes in microvascular endothelial cells through studying aorta endothelial cells. Therefore, it is useful to establish an *in vitro* microvascular endothelial cell culture system. In this study, we cultured myocardial microvascular endothelial cells and examined the purity using VIII related antigen immunohistochemical staining. Results showed that the purity of our microvascular endothelial cell culture was 95%, indicating that an *in vitro* microvascular endothelial cell culture system was successfully established. These microvascular endothelial cells may be used for cardiovascular disease therapy and clinical medicine research.

Endothelial cell proliferation is necessary for the formation of new vasculature. Cell proliferation can be negatively affected by two mechanisms: enhanced apoptosis and cell cycle progression arrest [20]. In this study, we tested the effect of various concentration of G-CSF on myocardium microvascular endothelial cells proliferation. We observed that addition of G-CSF increased the CMEC’s proliferative capacity. Result showed that CMEC’s proliferative capacity was the highest in the medium containing 40 ng/mL G-CSF. The mechanism was unclear. However, this study may provide the technology basis for future research of some related diseases. RT-PCR analysis showed that expression levels of HIF-1 and p53 mRNA were higher in the group treated with 40 ng/mL G-CSF than with the other two tested concentrations (20 ng/mL or 60 ng/mL). This indicated that 40 ng/mL G-CSF was the most effective dose for CMEC proliferation.

Hypoxia inducible factor-1 (HIF-1) is a potent α, β transcription factor that mediates tissue responses to hypoxia. Once activated, the heterodimer binds to the DNA consensus sequence 5′-RCGTG-3′, driving transcription of many genes involved in oxygen homeostasis, including inducible nitric oxide synthase (iNOS), vascular endothelial growth factor (VEGF), and heme oxygenase-1 (HO-1) [21–24]. It is well known that p53 plays an important role in controlling angiogenesis by different mechanisms, including regulation of vascular endothelial growth factor (VEGF) expression. Narasimhan *et al*. [25] reported that a mutant-type p53 could enhance VEGF expression induced by 12-*O*-tetradecanoylphorbol-13-acetate by activating protein kinase C. Cadwell and Zambetti [26] showed that wild-type p53 down-regulates VEGF promoter activity. This study showed that G-CSF treatment increased the HIF-1 mRNA expression level and reduced p53 mRNA expression in CMECs. G-CSF was significantly associated with VEGF expression. We suppose that G-CSF promoted angiogenesis partly through modulating HIF-1 mRNA expression, subsequently heightening cardiovascular compensation capacity. In addition, G-CSF still enhances CMEC’s proliferative capacity possibly partly through modulating p53 mRNA expression. We have searched a lot of relevant literature and do not find research regarding protein expression of p53 and HIF-1 in other systems. These hypotheses will be tested in future studies. Of course, a concrete mechanism still needs to be investigated in future studies.

## 5. Conclusion

In conclusion, our study showed that G-CSF promoted CMEC proliferation, possibly through modulating HIF-1 and p53 mRNA expression. This experimental data might provide a useful indicator of prognosis and therapy in heart diseases.
